# High Expression of SLC16A1 as a Biomarker to Predict Poor Prognosis of Urological Cancers

**DOI:** 10.3389/fonc.2021.706883

**Published:** 2021-09-22

**Authors:** Ling Zhang, Zheng-Shuai Song, Zhi-Shun Wang, Yong-Lian Guo, Chang-Geng Xu, Hao Shen

**Affiliations:** ^1^ Department of Pathology, Wuhan No.1 Hospital, Wuhan, China; ^2^ Department of Urology, The Central Hospital of Wuhan, Tongji Medical College, Huazhong University of Science and Technology, Wuhan, China

**Keywords:** SLC16A1, cancer in urology, generic cancer, biological marker, prognosis

## Abstract

**Objective:**

Tumor metabolism has always been the focus of cancer research. SLC16A1, as a key factor in catalysis of monocarboxylate transport across the plasma membrane, has been found to be associated with the occurrence and metastasis of a variety of cancers, but its prognostic significance and mechanism in different tumors are still unclear.

**Methods:**

Based on the gene expression matrix and clinical information of human cancer tissues acquired from TCGA and GTEX databases, the differential expression of SLC16A1 in different tumors and normal tissues was analyzed. To confirm the association between its expression, the mutation of MMRS gene, and the expression level of DNMTs. Univariate Cox regression was applied to analyze the association between SLC16A1 expression and patient prognosis. The effect of SLC16A1 expression on patient survival was examined by Kaplan Meier analysis. GSEA was used to identify related signaling pathways.

**Results:**

The expression of SLC16A1 was differentially expressed in most tumors, especially in the urinary tract where it is commonly highly expressed, and differential expression of SLC16A1 in different clinical stages. SLC16A1 expression was significantly positively correlated with MMRS gene mutation and DNMTS expression. Moreover, high SLC16A1 expression was associated with poorer overall survival (OS) and progression-free survival (PFS) in urological cancers. In particular, the results of the enrichment analysis showed that SLC16A1 was associated with processes such as cell adhesion and many signaling pathways affecting cell cycle were significantly enriched in the group with high-expressed SLC16A1.

**Conclusion:**

SLC16A1 expression was upregulated in urological cancer. SLC16A1 may promote tumor development by regulating the epigenetic process of urological cancer and demonstrated a great potential as a prognostic biomarker of urological cancer patients.

## Introduction

Urological cancer is a highly prevalent neoplastic disease with poor prognosis. Kidney cancer, bladder cancer and prostate cancer, which are the most common urological cancers, account for more than 33% of all male malignant tumors (1, 2) Different urological cancers show different morphological or genetic characteristics, which pose difficulties to the diagnosis and treatment in clinical practice (3, 4). Clinical symptoms of most urological cancers are non-specific, and a late diagnosis will increase the possibility of metastasis and adverse effects on the clinical outcome of patients (5). Although great progress had been made in the molecular study of urological tumors, the understanding of its pathological mechanism is still not satisfied (6). Identifying of certain genes and potential biological markers could improve the evaluation of the efficacy of late treatment (1) and the management of urological cancers.

Study demonstrated an important correlation among urinary system diseases, metabolic syndrome, and metabolic syndrome in endocrine system. Vascular mechanisms affect male urinary system, high insulin hematic disease, in which obesity is often involved. Therefore, discovering metabolism-related genes might provide the corresponding potential targets and improve the treatment of urinary system diseases (7). SLC16 family consists of 14 different monocarboxylate transporters, which play crucial roles in cell metabolism, nutrient transport, and pH regulation. Changes in the expression and function of some members are often reflected in serious diseases, such as cancer and nervous system diseases (8). SLC16A1 is a widely studied member of the SLC16A family. It has been found that SLC16A1 is distributed in almost all tissues in the human body, and is overexpressed in many cancers, moreover, upregulated expression of SLC16A1 is associated with the deterioration of prognosis of many cancers (9, 10).

Pan-cancer analysis is a novel bioinformatics method used to search commonalities across tumor types and organs, and could provide new adaptations for biomarkers across tumors (11). The Cancer Genome Atlas Project (TCGA) and Genotype Tissue Expression (GTEX) is currently the largest available databases for tumor genome analysis (12, 13). Based on TCGA and GTEX databases, this study applied pan-cancer analysis to detect the expression of SLC16A1 in different tumors and explored its prognostic significance. This study provided a potential molecular mechanism of the key role of SLC16A1 in urological cancer.

## Materials and Method

### Data Source

The TCGA database (https://www.cancer.gov/) contained gene expression profiles of tumors and adjacent normal tissues from different human cancers. In addition to GTEx (https://gtexportal.org/home/) and normal tissue expression, the TIMER 2.0 (http://timer.cistrome.org/) analysis on more than ten thousand samples of the RNA - seq TCGA database data was used and integrated into the TCGA expression. Gene expression matrices were obtained from the CCLE database (https://portals.broadinstitute.org/ccle) for cell lines of different origins, using the Human Protein Atlas (HPA) (https://www.proteinatlas.org/) database as well as the UALCAN (http://ualcan.path.uab.edu/) online website to validate the protein expression of SLC16A1 and its gene expression levels in different clinical features.

### Difference Analysis

Statistical differences in SLC16A1 expression levels between groups were calculated using the Wilcoxon test as well as the Kruskal-Wallis H-test. The chi-square test, as well as the t-test, were used to compare previous associations between gene expression and cultural characteristics and were presented visually using the R package ggplot.

### Correlation Analysis Between Gene Expression and Epigenetic Regulation

Epigenetic processes are considered to be an important factor affecting gene expression, and DNA replication and DNA methylation are markers in epigenetics (14). DNA mismatch repair (MMRs) plays crucial role in maintaining DNA replication and its structural integrity and stability (15). Changes in DNA methyltransferase (DNMTs) activity could affect gene expression and DNA repair mechanism, and dysfunction of MMRs and DNMTs is the initial conditions for human cancer development (16). Based on the expression profile of TCGA, Pearson test was performed to analyze the correlation between SLC16A1 expression and MMRS gene (MLH1, MSH2, MSH6, PMS2, EpCAM) mutation and DNMTs (DNMT1, DNMT2, DNMT3A, DNMT3B) expression. When R > 0.2 and P< 0.5, SLC16A1 expression was considered to be positively correlated with MMRS and methyltransferase expression.

### Survival Analysis

Univariate Cox regression analysis was performed on SLC16A1 expression level in different tumors using the survival package R, and 95% confidence intervals and corresponding hazard ratio (HR) were calculated. Forest plots were drew using the survival package Forestplot. Kaplan-Meier and timeROC was used to examine the relationship between SLC16A1 expression and the survival of urological cancer patients.

### Enrichment Analysis

According to the expression of SLC16A1, the samples from TCGA database were divided into high-expression and low-expression groups. Functional enrichment analysis was performed using the R package ClusterProfile based on Gene Ontology (GO) and the Kyoto Encyclopedia of Genes and Genomes (KEGG). A list of abs(log2FC) was generated based on two groups of high and low expression and this gene list was mapped GO, KEGG gene set for biological pathway analysis.

## Results

### Expression of SLC16A1 in Urological Cancers and Other Tumors

**Figure 1:**
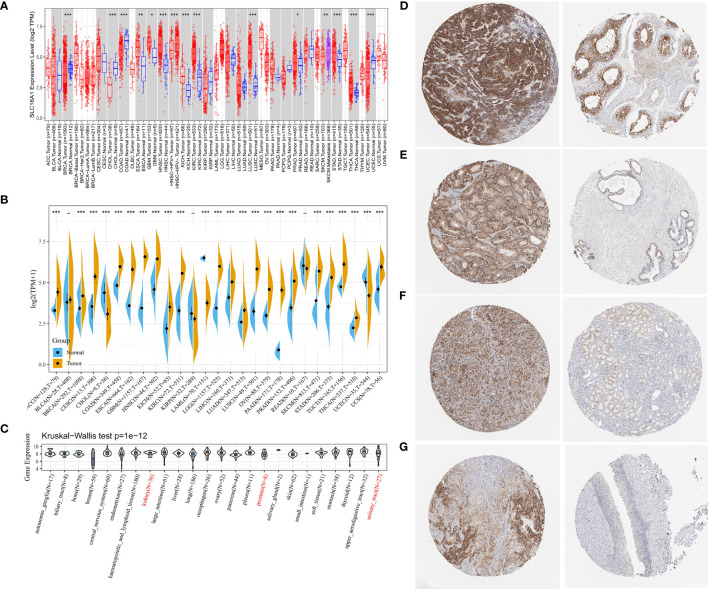
SLC16A1 expression levels in different types of human cancers. **(A)** Based on the TCGA database, the expression level of SLC16A1 between different cancers was analyzed using Timer 2.0; **(B)** The expression data of normal tissues in GTEX database were supplemented to analyze the expression levels of SLC16A1 in tumor tissues and normal tissues of different cancers. **(C)** SLC16A1 expression in each cell line obtained through the CCLE database. Immunohistochemical staining of SLC16A1 protein in **(D)** testicular cancer; **(E)** prostate cancer; **(F)** kidney cancer; **(G)** bladder uroepithelial cancer tissues and corresponding normal tissues was obtained from the HPA database. TCGA, Cancer Genome Atlas Project; GTEX, genotype tissue expression; HPA, Human Protein Atlas. ^*^P < 0.05, ^**^P < 0.01, ^***^P < 0.001.

We found that the expression level of SLC16A1 was different in most urological cancers (**Figure 1A**). The expression level of SLC16A1 in tumor tissues of BRCA, CHOL, COAD, UCEC and other cancer patients was lower than that in their corresponding paracancerous normal tissues (P<0.001). SLC16A1 expression was upregulated in metastatic SKCM when compared with carcinoma *in situ* (P<0.01). However, SLC16A1 was significantly overexpressed in tumor tissues of most other cancers (ESCA, GBM, HNSC, etc.) (P<0.05), and this feature was more strongly present in urinary tract tumors (Kich, Kirc, Prad) (P<0.05). However, normal samples in the TCGA database were generally few and even absent in some cancers. We also analyzed the normal samples in GTEX and tumor samples in TCGA and found that the high expression pattern of SLC16A1 was more obvious in urological cancers (ACC,KICH,Kirc,PRAD,TGCT) (**Figure 1B**). As shown in **Figure 1C**, SLC16A1 expression was present in cell lines of renal and prostate as well as bladder urogenital origin in the CCLE database. HPA for immunohistochemical staining on SLC16A1 protein in testicular cancer (**Figure 1D**), and prostate (**Figure 1E**) were found to have a high signal intensity, and this was also the same in Renal cancer (**Figure 1F**), uroepithelial cancer (**Figure 1G**). It is also shown that in comparison with the corresponding normal tissues, SLC16A1 was upregulated in the urinary diseases.

### Association Between SLC16A1 Expression and Clinical Features of Urological Tumors

**Figure 2:**
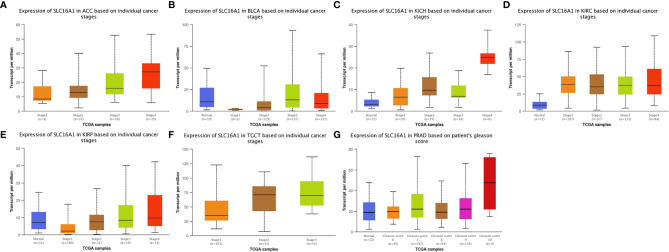
SLC16A1 expression correlates with different periods of urological cancer. SLC16A1 expression in **(A)** ACC, **(B)** BLCA, **(C)** KICH, **(D)** KIRC, **(E)** KIRP, **(F)** TGCT in different clinical stages and **(G)** PRAD in different Gleason.

To further compare the expression of SLC16A1 in urological cancers, patients were grouped according to clinical characteristics such as age, gender, race, TNM stage and clinical stage. As shown in **Table 1**, SLC16A1 overexpression was more prevalent in female patients in ACC, KICH, and KIRP, and that Caucasians had a higher proportion of SLC16A1 overexpression than other races in BLCA. High SLC16A1 expression was associated with higher T-staging in BLCA and KIRP, and with lymphatic metastases in ACC, KICH, KIRP, TGCT, and distant metastases in ACC and TGCT. Remarkably, SLC16A1 is closely associated with the clinical staging of most urological cancers. Differential expression of SLC16A1 in urological cancers of different clinical stages was also verified by the UALCAN database, as PRAD was missing clinical stage data, but significant differences in SLC16A1 expression were also found in patients with different Gleason scores, as detailed in **Figure 2**.

**Table 1:** Comparison of clinical characteristics of urological cancers patients with high and low expression of SLC16A1.

Clinical characteristics	Adrenocortical carcinoma			Bladder Urothelial Carcinoma			Kidney Chromophobe			Kidney renal clear cell carcinoma			Kidney renal papillary cell carcinoma			Prostate adenocarcinoma			Testicular Germ Cell Tumors	
	High expression	Low expression	P	High expression	Low expression	P	High expression	Low expression	P	High expression	Low expression	P	High expression	Low expression	P	High expression	Low expression	P	High expression	Low expression	P
Age		45.7 ± 17.0	47.7 ± 14.5)	0.576	68.8 ± 10.5	67.3 ± 10.7	0.154	53.6 ± 14.9	50.1 ± 13.2	0.321	60.1 ± 12.3	61.0 ± 12.0	0.394	62.6 ± 12.2	60.5 ± 11.6	0.136	61 ± 6.8	61.1 ± 6.9	0.871	30.1 (10)	33.9 (8.3)	/
Sex	Female	29	19		58	49		14	12		108	78		51	25		0	0		0	0	
	Male	11	20	**0.030**	146	155	0.311	19	20	0.685	157	187	**0.006**	93	119	**0.001**	248	248	/	67	67	/
Race	White	37	29		172	152		30	27		230	229		100	105		81	66		61	58	
	Asian	0	1		14	9		1	1		5	3		3	3		2	0		0	4	
	Black	0	1	0.292	9	35	**＜0.001**	1	3	0.565	26	30	0.675	33	27	0.698	6	1	0.130	3	3	0.131
TNM	T1	3	6		3	8		7	13		139	132		79	120		84	93		33	46	
	T2	19	23		85	106		14	11		28	41		23	8		97	107		31	19	
	T3	5	3		90	67		10	8		90	89		37	10		64	45		3	2	0.074
	T4	12	6	0.276	23	20	**0.043**	2	0	0.224	8	3	0.179	1	1	**＜0.001**	3	0	0.065	0	0	
	N0	30	38		121	116		18	21		115	124		66	77		173	171		36	44	
	N1~N3	9	0	**0.002**	67	62	0.872	5	0	**0.023**	7	9	0.735	22	5	**0.001**	41	38	0.797	22	8	**0.008**
	M0	27	35		80	116		27	23		225	215		93	112		231	223		60	66	
	M1	12	3	**0.011**	5	6	0.761	2	0	0.199	38	42	0.550	6	3	0.210	1	2	0.545	7	1	**0.029**
Stage	I	3	6		0	2		7	13	0.796	137	128		66	113		/	/	/	22	38	
	II	14	23		50	80		14	11		26	31		13	12		/	/	/	10	4	
	III	10	6		83	57		6	8		61	62		40	11		/	/	/	12	3	
	IV	12	3	**0.023**	70	64	**0.003**	6	0	**0.038**	39	43	0.814	12	3	**＜0.001**	/	/	/	22	20	**0.006**
Radiation	No	26	33		127	138		/	/	/	11	20		70	88		108	138		62	41	
	Yes	12	4	**0.028**	7	3	0.170	/	/	/	254	245	0.096	1	0	0.264	22	19	0.245	2	17	**＜0.001**
Neoadjuvant	Yes	1	0		6	4		/	/	/	8	9		144	144		1	1		0	0	
	No	39	39	0.320	198	200	0.522	/	/	/	257	256	0.805	0	0	/	247	247	/	67	67	/

Significant p values (p < 0.05) are in bold font.

### The Expression of SLC16A1 in Urological Cancers Promoted MMRS Mutations and DNMTs Activity

**Figure 3:**
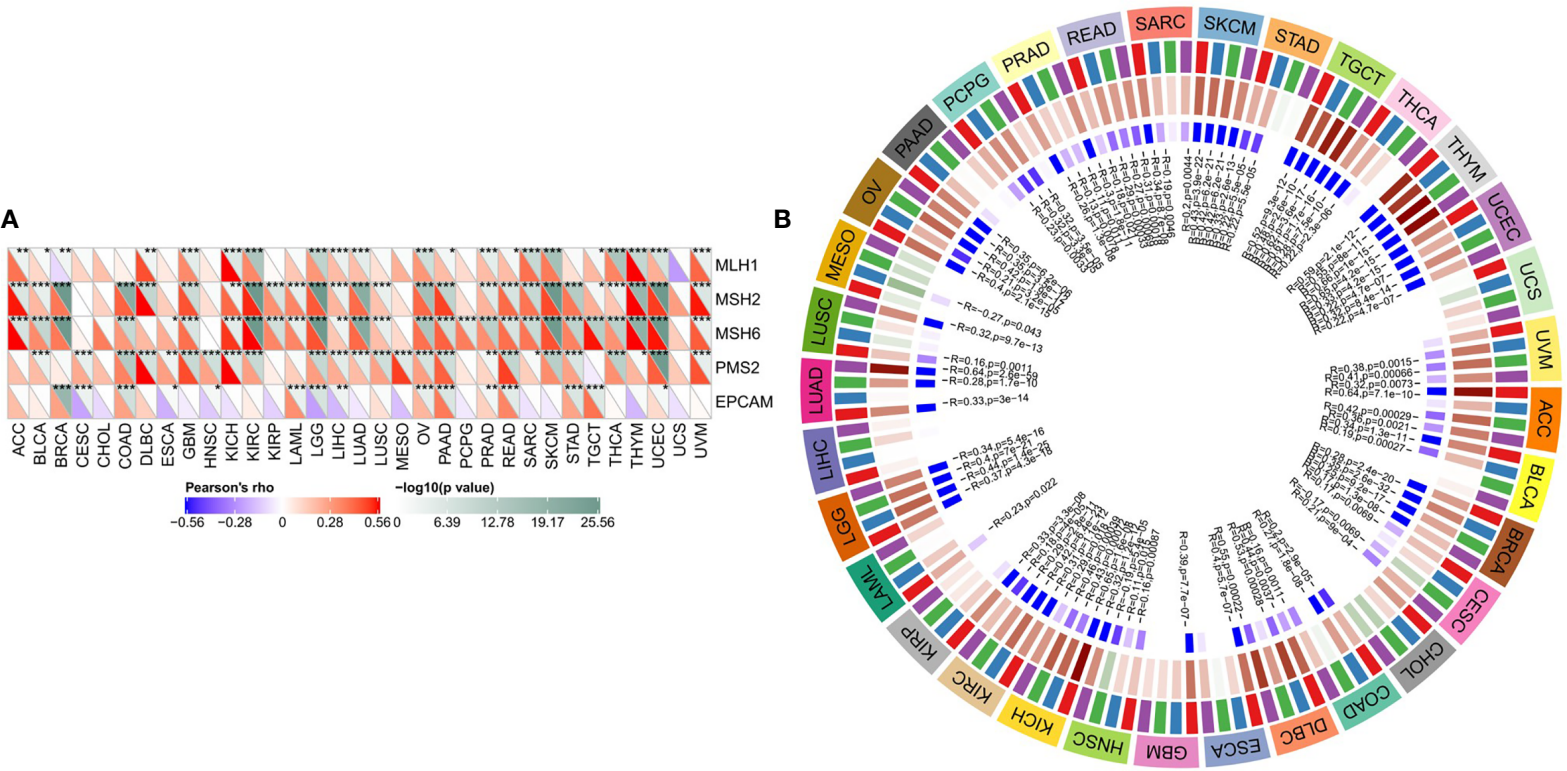
SLC16A1 expression in urological cancers promotes MMRS mutations and DNMTs activity. SLC16A1 expression and **(A)** ML H1, MSH2, MSH6, PMS2, EpCAM mutations; **(B)** Correlation between DNMT1, DNMT2, DNMT3A and DNMT3b expression. Note: MMRS: DNA mismatch repair; DNMTs: DNA methyltransferase. ^*^P < 0.05, ^**^P < 0.01, ^***^P < 0.001.

SLC16A1 expression was significantly positively correlated with gene mutations of MMRs in majority of tumors (**Figure 3A**). In particular, the three MMR genes (MLH1, MSH2, and MSH6) were significantly associated with almost all urinary tumors (ACC, BLCA, KICH, Kirc, Kirp, PRAD, TGCT). As shown in **Figure 3B**, SLC16A1 expression was positively correlated with DNMTs expression in all human tumors. KICH, Kirc, and Prad were significantly correlated with the expression of four DTMTs (**Figure 3B**). The results showed that SLC16A1 could promote the occurrence and development of tumor by inducing MMRS mutation and up-regulating the activity of DNMTs.

### High Expression of SLC16A1 Suggested a Poor Prognosis of Urological Cancers

**Figure 4:**
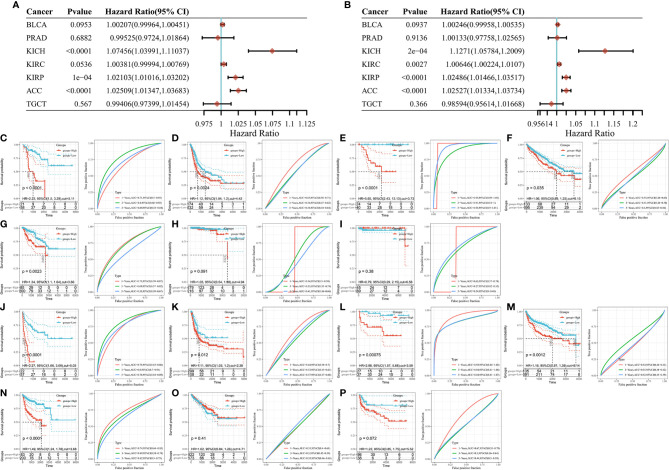
High SLC16A1 expression suggests a poor prognosis for urological cancer. Univariate Cox analysis of patients with different tumors **(A)** OS and **(B)** PFS; Kaplan Meier and timeROC analysis to detect the effect of SLC16A1 expression on OS in **(C)** ACC, **(D)** BLCA), **(E)** KICH, **(F)** KIRC, **(G)** KIRP, **(H)** PRAD, **(I)**TGCT patients and PFS in **(J)** ACC, **(K)** BLCA, **(L)** KICH, **(M)** KIRC, **(N)** KIRP, **(O)** PRAD, **(P)** TGCT patients. OS, total survival; PFS, progression-free survival; HR, Hazard ratio.

The association between SLC16A1 expression and overall survival (OS) in urological cancers was explored as shown in **Figure 4A**. SLC16A1 expression is a prognostic risk factor for ACC, KICH, and KIRP. To further analyze the impact of disease progression on prognostic outcomes, we also analyzed the effect of SLC16A1 expression on progression-free survival (PFS) of patients. SLC16A1 expression was significantly associated with ACC and PFS in the three renal cancers (**Figure 4B**). The prognostic value was also tested using Kaplan Meier as well as timeROC analysis. The results showed that overall survival was lower in urological patients with high SLC16A1 expression than in those with its low expression, except for two cancers, PRAD and TGCT (**Figures 4C–I**). Notably, SLC16A1 expression had a higher degree of impact on PFS in these urological patients (**Figures 4J–P**), and high SLC16A1 expression may suggest a poor prognosis for urological cancer patients.

### SLC16A1 Expression Was Associated With Carcinogenic Signaling Pathways

**Figure 5:**
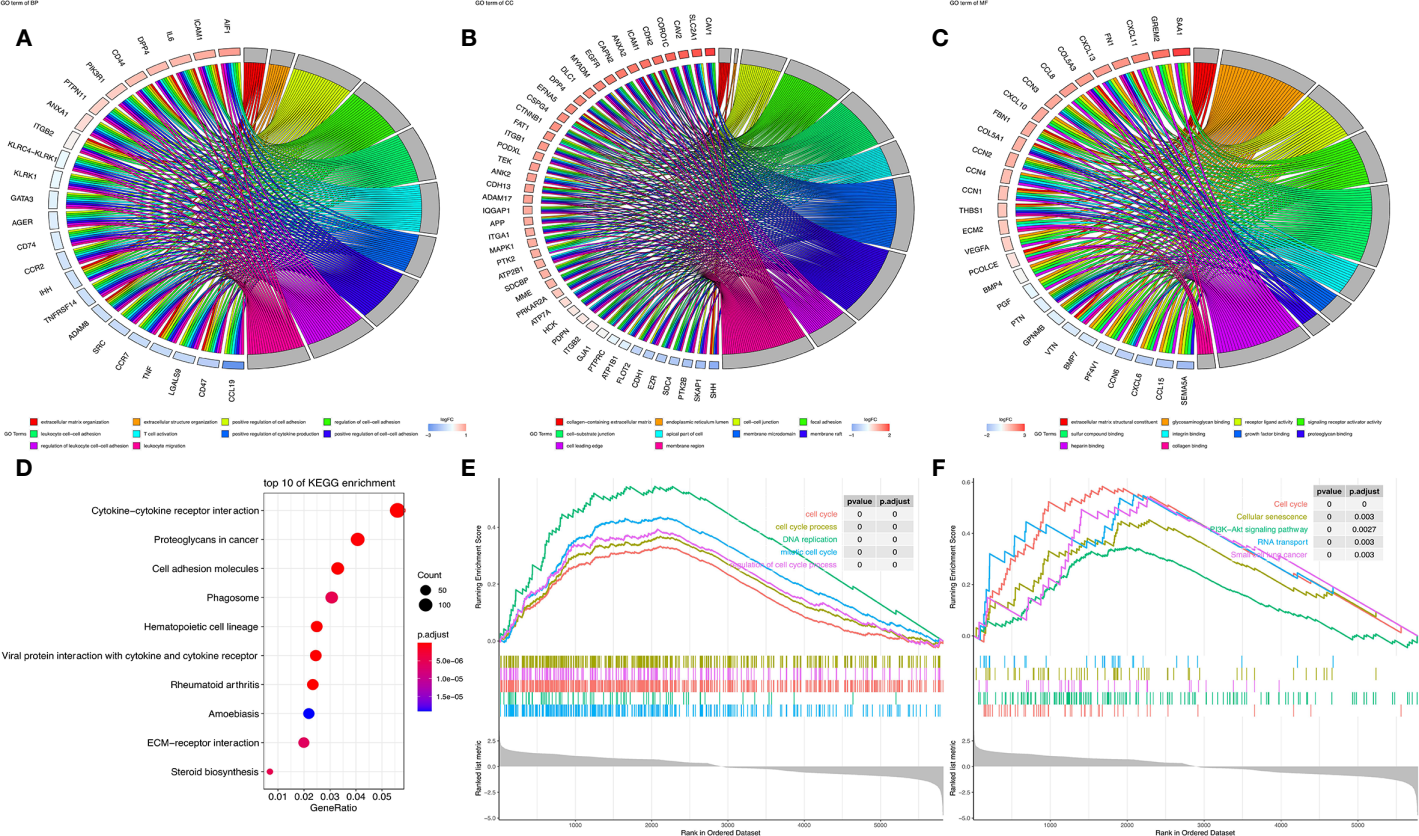
SLC16A1 expression is associated with carcinogenic signaling pathways. In GOChord plots showing the top 10 GO terms enriched in **(A)** BP, **(B)** CC, **(C)** MF and genes positively associated with SLC16A1; **(D)** bubble plots to show the top 10 pathways in KEGG analysis; GSEA analysis showing the set of **(E)** GO and **(F)** KEGG genes associated with SLC16A1 expression. GSEA, single gene set enrichment analysis; GO, Gene Ontology; BP, Bioengineering; CC, Cellular Component; MF, Molecular Function; KEGG, Kyoto Encyclopedia of Genes and Genomes.

GOChord diagram visualizing the interconnection between GO terms and genes. As shown in **Figure 5A**, SLC16A1 was associated with cell activation, biological processes such as cell adhesion (BP), and was significantly enriched in intercellular-related cellular components such as focal adhesion (CC) (**Figure 5B**) and binding-related molecular functions such as glycosaminoglycan binding (MF) (**Figure 5C**). The results of KEGG analysis showed that slc16a1 expression was correlated with signals including cytokine cytokine receptor interaction, proteoglycans in cancer, and cell adhesion molecules. Using GSEA to look for signals that are activated in cancer, the most significantly enriched GO and KEGG signaling pathways have been listed in the top right corner of **Figures 5E, F**. High SLC16A1 expression was associated with cell cycle, PI3K-Akt and other signaling pathways, which may suggest that high SLC16A1 expression may be involved in tumor progression through regulation of the cell cycle.

## Discussion

Tumor cells show a much higher growth activity than normal cells. In recent years, it has been suggested that tumor cells metastasize to escape metabolic stress, which is a process that often involves metabolic reprogramming. Rewriting of energy metabolism pattern will confer tumor cells the ability to escape normal apoptotic process and to grow, proliferate and migrate (17). Since Warburg found that cancer cells tend to produce energy through glycolysis, many studies have been carried out on the energy metabolism of tumors. Studies have found that cancer cells still obtain energy through glycolysis despite the presence of oxygen (18). Lactic acid as the main product of anaerobic metabolism is considered to be one of the most important energies. The continuous production and accumulation of lactic acid can lead to metabolic gene expression changes, result in abnormal cell signal, weaken the function of mitochondria, upregulate SLC16 protein expression, thereby leading to cell migration and immune escape, and the promotion of cancerous cell process (19). SLC16A1 plays an important role in cancer metabolism. Lactic acid itself can rapidly upregulate SLC16A1 expression, which maintains glycolysis efficiency by regulating pH value in cells and interstitium. SLC16A1 plays a major role in lactate inflow, and cancer cells transport lactic acid inward through overexpression of SLC16A1, increasing carcinogenicity and invasion. Thus, targeting SLC16A1 may be a promising therapeutic strategy for some cancers (20, 21).

Based on TCGA and GTEX databases, we compared SLC16A1 expression in 27 different human cancers using TIMER and differential analysis. The results showed that SLC16A1 was differentially expressed in most solid cancers, particularly concentrated in urological cancers. This is also supported by the results of the CCLE database: SLC16A1 is significantly expressed in cell lines of urological origin. Further analysis of SLC16A1 expression in urological cancers showed that SLC16A1 protein expression was significantly stronger in pathological tissues of the kidney, prostate, and bladder than in the corresponding normal tissues, and that there was an association between high and low SLC16A1 expression and the different clinical features of urological cancers. SLC16A1 was differentially expressed between the different clinical stages of seven different urological cancers, and it is therefore conjectured that SLC16A1 may be involved in the disease progression of urological cancers.

Aberrations in epigenetic mechanisms have now been recognized as an important cause of human cancer progression (22). The continuous accumulation of MMRS gene mutations throughout the genome will allow the malignant transformation of cell signals, and promote the carriers of MMRS mutation to develop cancer (23). DNA methylation is critical for maintaining cellular phenotypes during DNA replication and is involved in defining when and where genes are expressed in normal and disease environments (24, 25). Currently, DNMTs inhibitors are the most widely studied compounds that inhibit epigenetic processes and have also been used in the treatment of cancer (26). Our results showed that SLC16A1 expression was significantly positively correlated with MMRs mutation and expression of DNMTs in urological tumors, indicating that SLC16A1 was related to the occurrence and development of urological tumors.

Besides, high expression in urological cancers appears to be with an increased prognostic risk. Our study found that SLC16A1 could serve as a biomarker for the prediction of OS and PFS of urological cancers and identifying cancer subtypes with higher prognostic risk, especially in several cancers of ACC, KIRC and KICH. SLC16A1 plays a key role in promoting cancer progression and metastasis. It has been found that SLC16A1 and SLC16A1-AS1 may together form a “head-to-head” complex unit with E2F1 promoter in muscle-infiltrating bladder cancer cells. E2F1 activates SLC16A1 and SLC16A1-AS1 to cooperatively regulate the corresponding targets related to cell migration and promote metabolic reprogramming and cell migration (27). High-expressed SLC16A1 has also been found to be associated with lymph node metastasis and distant metastasis of bladder cancer, thereby showing a negative impact on the overall survival of patients. Inhibiting SLC16A1 can significantly suppress the proliferation, migration and invasion of bladder cancer cells, and SLC16A1 promotes the progression of bladder cancer by affecting epithelial mesenchymal transformation and glycolysis (28). Fuhrman grade is a histological grade of renal cancer based on tumor nuclear morphology, and has been widely applied as the most effective prognostic parameter for predicting DSS (29). Ambrosetti et al. (30) conducted a semi-quantitative and qualitative analysis on 45 Kirc cases, and found that SLC16A1 is positively correlated with higher Fuhrman grade. All the above studies suggested that SLC16A1 is a risk factor for evaluating the prognosis of urological cancers.

On the other hand, to further study the role of SLC16A1 in tumors, We found by enrichment analysis that SLC16A1 expression is associated with cell activation, cell adhesion and other functions, and may be involved in signaling pathways such as the cell cycle. During tumor progression, metabolic reprogramming is often accompanied by cytoskeletal remodeling and the activation of transduction of mechanical signaling in cancer cells. Cancer cells induce metabolism-related signaling by continuously altering intercellular adhesions and finally by glycolysis to meet the increased motility as well as aggressiveness of cancer cells (31, 32). We speculate that the high expression of SLC16A1 in urological cancers may also suggest a metabolic reprogramming mediated by altered intercellular adhesion.

In Pereira’s study, to maintain a high glycolytic phenotype, prostate cancer is effectively transported to the breast *via* SLC16A1 and SLC16A4.The expression level of SLC16A1 in metastatic prostate cancer cells is significantly higher than that in more restricted prostate cancer cell lines. Silencing SLC16A1 can significantly inhibit the growth and motor characteristics of cancer cells (33), high expression of SLC16A1 may stimulate prostate cancer cell activation. Feng et al. (34) also speculated that SLC16A1-AS1 might be involved in the occurrence and metastasis of cancer through regulation of cell cycle through three different algorithms of WGCNA, GSEA and GSVA, and verified at the cellular level that SLC16A1-AS1 silencing could inhibit the expression of cyclin D1, promote cell stagnation in G0/G1 phase, and suppress the proliferation of oral squamous cell carcinoma cells. In Addition, we also found that SLC16A1 is associated with signaling pathways such as P13K-Akt, which is also a key factor in the cell cycle, and targeting PI3K-Akt signaling may induce cell cycle arrest (35, 36). This also suggests that SLC16A1 is involved in tumor development from another perspective.

To sum up, this research showed that SLC16A1 expression was present in urological tumors and found that the high expression of SLC16A1 was related to poor prognosis of patients with urological cancer and abnormal epigenetic processes, providing clinically useful biological markers.

## Data Availability Statement

The original contributions presented in the study are included in the article/supplementary material. Further inquiries can be directed to the corresponding author.

## Author Contributions

HS, Z-SS, Z-SW, Y-LG, C-GX, and LZ: These authors have contributed equally to this work. All authors contributed to the article and approved the submitted version.

## Funding

This study was supported by Wuhan Health Research Foundation (No.WX21M04), National Natural science Foundation of China (Grant No. 82002722), Hubei Province Health and Family Planning Scientific Research Project (No.WJ2018H209), Natural Science Foundation of Hubei Province (No.2020CFB175), and Research Fund of Wuhan Health and Family Planning Commission (No.WX20Q24).

## Conflict of Interest

The authors declare that the research was conducted in the absence of any commercial or financial relationships that could be construed as a potential conflict of interest.

## Publisher’s Note

All claims expressed in this article are solely those of the authors and do not necessarily represent those of their affiliated organizations, or those of the publisher, the editors and the reviewers. Any product that may be evaluated in this article, or claim that may be made by its manufacturer, is not guaranteed or endorsed by the publisher.
